# Assessment of Two Different Drinking Water Treatment Plants for the Removal of Free-living Amoebae, Egypt

**Published:** 2017

**Authors:** Ahmad Z. AL-HERRAWY, Mahmoud A. GAD

**Affiliations:** Dept. of Water Pollution Research, National Research Center, 12622 Dokki, Giza, Egypt

**Keywords:** Removal, Efficacy, Free-living amoebae, Drinking water treatment plants, PCR

## Abstract

**Background:**

The aim of this study was to compare between slow and rapid sand filters for the removal of free-living amoebae during drinking water treatment production.

**Methods:**

Overall, 48 water samples were collected from two drinking water treatment plants having two different filtration systems (slow and rapid sand filters) and from inlet and outlet of each plant. Water samples were collected from Fayoum Drinking Water and Wastewater Holding Company, Egypt, during the year 2015. They were processed for detection of FLAs using non-nutrient agar (NNA). The isolates of FLAs were microscopically identified to the genus level based on the morphologic criteria and molecularly confirmed by the aid of PCR using genus-specific primers.

**Results:**

The percentage of removal for FLAs through different treatment processes reached its highest rate in the station using slow sand filters (83%), while the removal by rapid sand filter system was 71.4%. Statistically, there was no significant difference (*P*=0.55) for the removal of FLAs between the two different drinking water treatment systems. Statistically, seasons had no significant effect on the prevalence of FLAs in the two different drinking water treatment plants. Morphological identification of the isolated FLAs showed the presence of 3 genera namely *Acanthamoeba*, *Naegleria*, and *Vermamoeba* (*Hartmannella*) confirmed by PCR.

**Conclusion:**

The appearance of FLAs especially pathogenic amoebae in completely treated drinking water may cause potential health threat although there is no statistical difference between the two examined drinking water filtration systems

## Introduction

Water contamination is a common problem to all over the world (1). The microbial contaminants include pathogens like bacteria, viruses, and parasites such as microscopic protozoa and worms. Human and animal wastes knowing or unknowingly can spread these living organisms (2). Moreover, free-living amoebae (FLAs) have the ability to survive in diverse environments and have been isolated from soil, different aquatic environments and even air, indicating the ubiquitous nature of these organisms (3–7). In addition, FLAs have been detected and consequently isolated from hospitals (like dialysis units, eyewash stations) and clinical samples (human lungs tissues, nasal cavities, corneal biopsies, pharyngeal swabs, skin lesions, brain cerebrospinal and fluid necropsies) (8–10). Unlike “true” parasites, pathogenic FLAs can complete their life cycles in the environment without entering a human or animal host. Some of FLAs are pathogenic for humans (11). Most *Acanthamoeba* species have an association with human disease as granulomatous amoebic encephalitis (GAE), pulmonary and kidney infections, nasopharyngeal, cutaneous lesions, primarily in immunocompromised patients. *Acanthamoeba* species also cause amoebic keratitis in immunocompetent persons. Another species, *Balamuthia mandrillaris* close relative to *Acanthamoeba*, cause skin and lung infections as well as fatal GAE mostly in healthy children. *Naegleria fowleri* causes a non-opportunistic primary amoebic meningoencephalitis (PAM) in healthy children and young adults. *Sappinia pedata* has been reported from a brain infection in a healthy man (12). *Vahlkampfia, Vannella,* and *Vermamoeba* species have also been isolated from the eye surface of humans (13, 14).

Free and combined chlorine at 10 mg L^−1^ was reported to be effective against *Hartmannella vermiformis* cysts after 30 min exposure (15), but it is clearly ineffective for acanthamoeba cysts because they can resist exposure to 50 mg L^−1^ for 18 h (16) or 100 mg L^−1^ chlorine for 10 min (17). Two log reduction of *Naegleria* cysts could be achieved by chlorine with CT value 29 mg min /L (18).

The act of producing drinking water free from waterborne pathogens is considered the main objective of water treatment providers. Because no single treatment process can be expected to remove all of the different types of pathogens found in water, multiple barriers (pre-chlorination, coagulation, and sedimentation, filtration and post-chlorination) are desirable. Filtration is a physical removal of organisms together with other particulate matter. Various filtration processes (as rapid and slow sand filtrations) are used in conventional drinking water treatment plants (19). In Egypt, although rapid sand filters are widely used in conventional drinking water treatment plants, the slow sand filters are also used but in a small scale.

Therefore, the aim of this study was to compare between a slow sand filter and a rapid sand filter in the corresponding drinking water treatment plants for removal of FLAs and to identify the isolated free-living amoebae.

## Materials and Methods

### Operational design of a drinking water treatment plant

This study was conducted on two different drinking water treatment plants (DWTPs) located in Fayoum Drinking Water and Wastewater Holding Company, Egypt during 2015. One DWTP was operated by rapid sand filtration system, while the other was operated by slow sand filtration system. Moreover, rapid sand filters required smaller land areas compared to slow sand filters, so they were widely used in large municipal water systems by the 1920s. Rapid sand filters use relatively coarse, sand and other granular media to remove impurities and particles trapped in a flow through the use of chemicals—typically alum for flocculation. After flocculation step, the unfiltered water flows through the filter medium under pumped pressure and the floc material is trapped in the sand matrix. With respect to slow sand, filtration is a process involving passage of raw water through a sand bed at low velocity (generally less than 0.4 m/h) compared with 20 m/h in a rapid granular media filtration, resulting to substantial particulate removal by physical and biological mechanisms. These filters work through the formation of a gelatinous layer (or biofilm) named *Schmutzdecke*. This layer laid at the top few millimeters of the fine sand layer. *Schmutzdecke* is formed in the first 10–20 d of operation and consists of fungi, bacteria, protozoa, rotifer and a range of aquatic insect larvae. *Schmutzdecke* layer provides the effective purification in potable water treatment; sand underlying *Schmutzdecke* layer provides the support medium for this biological treatment layer. Then water passes through the hypogeal layer, foreign matter particles are trapped in the mucilaginous matrix and soluble organic material is adsorbed. Microorganisms as the bacteria, fungi, and protozoa metabolized contaminants (20, 21).

**Fig. 1:**
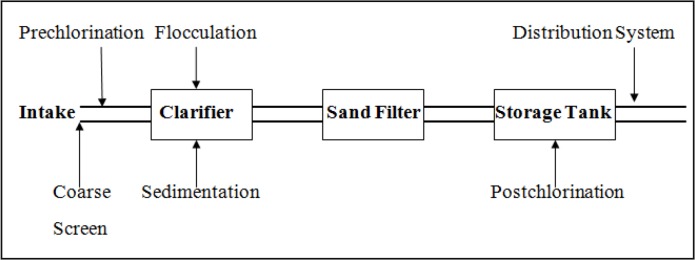
Operating diagram for a conventional drinking water treatment plant

A conventional drinking water treatment plant consists of 4 different steps beginning from the intake water (raw surface water). Raw water from the intake is sucked in pipes having coarse metal sieves with 4cm pore size for prevention of coarse objects from getting entrance with sucked water. The sieved raw water is pumped to coagulation and precipitation basins where it is mixed with aluminum sulfate to aid in the flocculation and precipitation of the debris and microorganisms found in raw water. After that, the clear water in the top of sedimentation basins is collected and passed on sand filters to get rid of the remaining microorganisms as well as escaped very small particles. Filtered water is collected in storage tanks where it is injected with chlorine dose of 2mg/l for disinfection. The disinfected water (outlet water) is ready to be pumped and distributed to the consumers as a drinking water (Fig. 1) (21).

### Water samples

Two types of water were collected from the two previously mentioned DWTPs: raw (inlet) water and treated (outlet) water. Water samples (one-liter volume each) were collected monthly along on year from each of the two DWTPs. The samples were separately collected in autoclavable polypropylene containers (one-liter volume) (22).

### Concentration, cultivation and morphological characterization of freshwater amoebae from collected samples

Collected water samples were separately concentrated by using the membrane filtration technique. Each water sample (One-liter volume) was filtered through a nitrocellulose membrane filter (0.45 μm pore size and 47mm in diameter) by using a stainless steel holder connected with a suction pump. The membrane of each filtered water sample was face to face inverted on the surface of non-nutrient agar medium seeded with living *E. coli* bacteria and incubated at 30 °C for two week with daily microscopic examination using the inverted microscope (22). Plates proved to have FLAs were sub-cultured and cloned on new NN agar plates seeded with *E. coli* for further morphological and molecular analysis. All cloned amoebae were evaluated with morphological criteria according to page key (23).

### DNA extraction and Polymerase Chain Reaction

Cloned plates were washed with sterile PBS buffer. FLAs were then centrifuged at 250 xg for 5–10 min. Amoebic DNA extractions for amoebae were performed using a modified phenol-chloroform method (24) and modified (25).

The PCR reaction was done using four different primer pair for *Acanthamoeba*, *Vermamoeba vermiformis*, *Naegleria* and *N. fowleri*. Presence of *Acanthamoeba* was confirmed by genus specific primer pairs AcantF900 (5′-CCCAGATCGTTTACCGTGAA-3′) and AcantR1100 (5′-TAAATATTAATGCCCCCAACTATCC-3′) which could amplify 18S rRNA gene (26). Primers, Hv1227F (5-TTACGAGGTCAG GACACTGT-3) and Hv1728R (5-GACCATCCGGAGTTCTCG-3) were used for amplify18S rDNA of *Vermamoeba* (*Hartmannella*) *vermiformis* (27, 28). *Naegleria* was identified by genus specific primer (5-CAAACACCGTTATGACAGGG-3) and (5-CTGGTTTCCCTTACCTTGCG-3) (28). In addition, species-specific primer was used to confirm the presence of *Naegleria fowleri* (5-GTGAAAACCTTTTTTCCATTTACA-3) and (5-AAATAAAAGATTGACCATTTGAAA-3) (29). Amplification of DNA was performed using Maxima™ Hot Start Green PCR Master Mix (Thermo Fisher Scientific Inc, Waltham, MA, USA) according to the manufacturer manual. PCR reaction mixture used per sample consisted of 25 μL Maxima Hot Start Green PCR Master Mix, 3 μL template DNA, 1 μL of each primer, and 20μL diethylpyrocarbonate (DEPC)-treated water. The DNA was visualized using ethidium bromide.

The obtained data were analyzed by one-way ANOVA, two samples t-test and Paired t-test using Minitab statistical program. A *P*-value <0.05 was considered significant (30).

## Results

### Prevalence of FLAs in rapid sand filtration system drinking water treatment plant (RSFS DWTP)

**Fig. 2:**
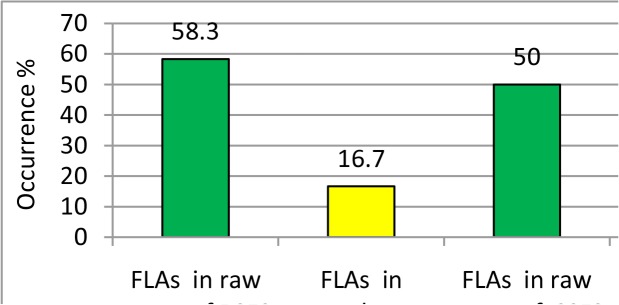
Occurrence of FLAs in the examined DWTPs

The occurrence of FLAs (FLAs) in water samples collected from the intake of RSFS DWTP reached 58.3%, while the lowest occurrence was observed in treated water samples (16.7%). By conventional statistical criteria, the removal of FLAs by RSFS DWTP is considered to be significant (*P*=0.017) by Paired *t*-test (Table 1, Fig. 2).

**Table 1:** Prevalence of FLAs in RSFS DWTP

***Sample types***	***Total number of examined samples***	***FLAs-positive samples on NN agar No.***	***%***
**Raw water**	12	7	58.3
**Treated water**	12	2	16.7

### Prevalence of FLAs in slow sand filtration system drinking water treatment plant (SSFS DWTP)

The occurrence of FLAs in the samples collected from the intake of SSFS DWTP reached 50.0%. Consequently and after complete water treatment, the occurrence of FLAs decreased to be 8.3% in treated water samples. Statistically, the removal of FLAs by SSFS DWTP is considered to be significant (*P*=0.017) by Paired t-test (Table 2, Fig. 2).

**Table 2:** Occurrence of FLAs in SSFS DWTP

***Sample types***	***Total number of examined samples***	***FLAs-positive samples on NN agar***			
		No.	%
Raw water	12	6	50.0
Treated water	12	1	8.3

The occurrence of FLAs in intake of RSFS DWTPs was higher (58.3%) than that in intake of SSFS DWTP (50%). In addition, the presence of FLAs was higher (16.7%) in treated water of RSFS DWTP than in treated water of SSFS DWTP (8.3%) (Fig. 2).

### Seasonal variation of FLAs in water of the examined DWTPs

Concerning seasonal variations, the highest occurrence of FLAs in the intake of RSFS DWTP was recorded in summer (100%), followed by 66.7% in spring. The same occurrence percentage of FLAs (33.3%) was recorded in winter and autumn for each. Concerning intake of SSFS DWTP, the percentage of occurrence of FLAs were the same (66.7%) in summer and autumn, while the occurrence was lowered to be 33.3% in both winter and spring. Statistically, *P*=0.67 (i.e. higher than 0.05) so seasons had no significant effect on the prevalence of FLAs in raw water of the two DWTPs. In treated water of RSFS DWTP, FLAs were detected in percent 33.3% in each of summer and autumn. In treated water of SSFS DWTP, the FLAs were recorded only in summer season in percent 33.3%. Statistically, *P*=0.537, therefore, seasons had no significant effect on the prevalence FLAs in treated water of two different DWTPs by using one-way ANOVA (Table 3).

**Table 3:** Seasonal variation of FLAs in the examined DWTPs

***Season***	***FLAs-positive Samples on NN agar in RSFS DWTP***						***FLAs-positive Samples on NN agar in SSFS DWTP***					
	Intake water samples			Finished water samples			Intake water samples			Finished water samples		
	No. of collected samples	No.	%	No. of collected samples	No.	%	No. of collected samples	No.	%	No. of collected samples	No.	%
**Winter**	3	1	33.3	3	0	0	3	1	33.3	3	0	0
**Spring**	3	2	66.7	3	0	0	3	1	33.3	3	0	0
**Summer**	3	3	100	3	1	33.3	3	2	66.7	3	1	33.3
**Autumn**	3	1	33.3	3	1	33.3	3	2	66.7	3	0	0

### Efficiency of DWTPs for the removal of free-living amoebae

The removal percentage of FLAs through different treatment processes reached its highest rate in SSFS DWTP (83%), while the removal of FLAs by RSFS DWTP was decreased to 71.4% (Table 4).

**Table 4:** Efficiency of DWTPs for the removal of FLAs

	***FLAs (occurrence %)***		
**DWTPs**	Raw water	Treated water	FLAs removal (%)
**RSFS**	7	2	71.4
**SSFS**	6	1	83.0

Statistically (*P*=0.55), there was no significant difference for the removal of FLAs between two different drinking water treatment plants by using 2-sample t-test.

### The occurrence of different genera of FLAs in DWTPs

**Fig. 3:**
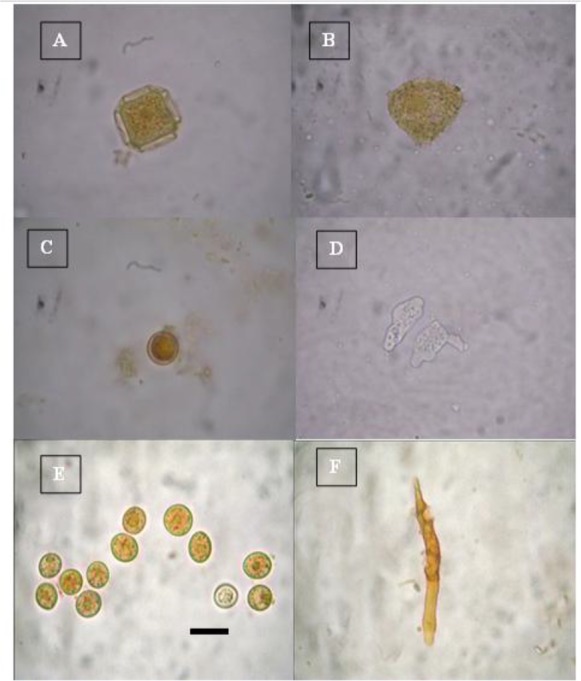
A: *Acanthamoeba* species cyst stained with Lugol’s iodine. B: *Acanthamoeba* species trophozoite stained with Lugol’s iodine. C: *Naegleria* species cyst stained with Lugol’s iodine. D: Unstained *Naegleria* species trophozoites. E: *Vermamoeba* species cysts stained with Lugol’s iodine. F: *Vermamoeba* species trophozoite stained with Lugol’s iodine. Bar = 10 μm.

Examination of the collected water samples from two different DWTPs revealed the isolated FLAs related to 3 genera (*Acanthamoeba, Naegleria*, and *Vermamoeba*)*.* In addition, *Acanthamoeba* cysts had different shapes. *Acanthamoeba* spp. were isolated from six samples collected from inlet of RSFS DWTP and five samples collected from inlet of SSFS DWTP. In addition, genus *Acanthamoeba* was detected in two samples collected from the outlet of RSFS DWTP, while it was detected in one sample collected from the outlet of SSFS DWTP. Members of genus *Naegleria* was isolated from one sample of inlet of RSFS DWTP, but they did not appear in other water samples collected from DWTPs. *Vermamoeba vermiformis* was isolated only from one inlet water sample of SSFS DWTP, but it did not appear in other water samples (Table 5, Fig. 3).

**Table 5:** Distribution of the isolated FLAs in sampling sites of DWTPs

***FLAs***	***Sampling site***							
	***RSFS DWTP***				***SSFS DWTP***								
	***Inlet***		***Outlet***		***Inlet***		***Outlet***	
	***Morphology***	***PCR***	***Morphology***	***PCR***	***Morphology***	***PCR***	***Morphology***	***PCR***
***Acanthamoeba* spp.**	6	6	2	2	5	5	1	1
***Naegleria* spp.**	1	1	0	-	0	-	0	-
***Vermamoeba vermiformis***	0	-	0	-	1	1	0	-

Means not tested

### Molecular characterization of the isolated free-living amoebae

**Fig. 4:**
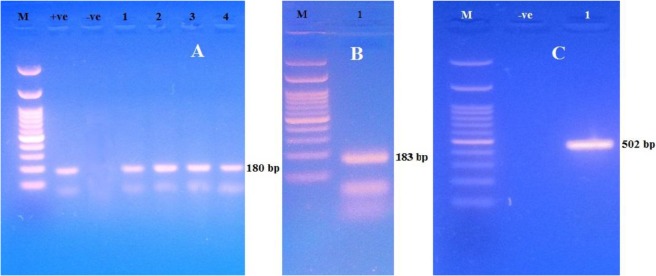
Agarose gel electrophorisis for PCR amplified product of DNA from (A) *Acanthamoeba* spp. Lane 1: 100 plus DNA ladder; Lane 2: Control Positive; Lane 3: Control negative; lanes 4, 5, 6 and 7: Positive samples (1–4). (B): *Naegleria* spp. Lane 1: 100 plus DNA ladder; lane 2: Positive sample. (C): *Vermamoeba vermiformis*. Lane 1: 100 plus DNA ladder; lane 2: Negative control; lane 3: Positive sample

The morphologically identified FLAs were subjected to molecular confirmation by simple PCR techniques using genus specific primers for *Acanthamoeba,* and *Naegleria* as well as species-specific primers for *N. fowleri* and *Vermamoeba vermiformis.* All morphologically identified *Acanthamoeba* strains proved to be related to genus *Acanthamoeba* when they were tested by PCR. In addition, morphologically *Naegleria-*positive sample proved to be related to genus *Naegleria* by PCR. On the other hand, *N. fowleri* amoebae were not detected by PCR in *Naegleria*-positive samples. The microscopically *Vermamoeba*-positive sample gave a specific band for *V. vermiformis* (Table 5, Fig. 4).

## Discussion

The act of safe drinking water production is of a public health concern worldwide. The removal percentage of FLAs through different treatment processes reached its highest rate in SSFS DWTP (83%), while in RSFS DWTP it was decreased to 71.4%. The treatment processes applied to different stages of drinking water treatment production (including RSFS) in Damanhour DWTP, Behera governorate, could remove 75% of FLAs present in the inlet water (7). Moreover, in Egypt, The highest removal efficiency (72.7%) was recorded in New Azab DWTP (using RSFS), followed by Old Azab DWTP (using RSFS) (70%) (31). At the same time, both New Kohafa (using SSFS) and Old Kohafa DWTPs (using RSFS) recorded the same removal efficiency representing 57.1% for each. These results were nearly similar to our results especially the elimination of FLAs by RSFS DWTP. There are factors known to affect the presence of FLAs, like water source, water treatment method and geographic location (32). In our opinion, the higher removal of FLAs from SSFS DWTP was an indication that treatment steps were more efficiently processed than that of the examined RSFS DWTP. Recently, the concentration of the disinfectant and contact time (Ct) values (in mg min/L) required for 2-log reduction of *Acanthamoeba, Naegleria* and *Vermamoeba* cysts treated with chlorine reached 865, 29 and 156, respectively (18). In our opinion, the chlorine doses (2–7 mg/L) actually used for disinfection of the produced water in drinking water treatment plants were insufficient for getting rid of free-living amoebae.

The present investigation showed that the removal of FLAs by slow sand filtration was better than that by rapid sand filtration. The capability of slow sand filters in getting rid of living organisms from drinking water was clearly discussed (33). A slow sand filter was first put into operation, a bio-layer called a *Schmutzdecke* and made of exocellular polymers (complex proteins and carbohydrates) was produced on the top slow sand filter as a result of accumulation and subsequent growth of aquatic aerobic microscopic organisms and living organisms consisting of algae, diatoms, bacteria, and zooplankton. This sand and bio-layer must always be submerged under oxygen rich water, and it was very effective at mechanically filtering very small particles out of the water flowing through it. In addition, the living organisms in the bio-layer literally eat pathogens in the water that are caught in the bio-layer from a process known as biological flocculation (they stick to the biofilm). The sandy column under the bio-layer acted as a mechanical filter for water passing through. “Moreover, the small aquatic organisms grown in the bio-layer can produce toxic substances for pathogenic viruses and bacteria present in the flowing water through the filter” (33, 34).

Slow sand filtration systems in drinking treatment plants can give some level of protection against pathogenic microorganisms. Different studies confirmed a pronounced level of elimination of microorganisms as protozoa and bacteria through slow sand filtration systems. Once a microbiological population was established after two weeks within the sand bed, the removal of total coliforms increased to 4 logs and no *Giardia* was detected in the filtered water (20).

Concerning seasonal variations in the present work, it was observed that FLAs prevailed in the warm seasons. In another study in Egypt, it was found that the highest occurrence of FLAs in the raw water of the examined DWTPs was recorded in summer (91.7%), followed by spring, autumn and winter in percentages 83.3, 75 and 41.6%, respectively (31). The results of Al-Herrawy et al. (31) were in concordance with the results of the present study. *Acanthamoeba* occurred in freshwater samples in a percentage of 33.3% all over the year (7). In our opinion, abundance of FLAs may be greatly affected by the location of sampling. FLAs in faucet water were uniformly circulated in both spring and fall (16.7% for each), while they prevailed in of Greater Cairo in winter (41.7%), trailed by summer (25%) (35).

FLAs in faucet water were uniformly circulated in both spring and fall (16.7% for each), while they prevailed in of Greater Cairo in winter (41.7%), trailed by summer (25%).

In the USA, the increased occurrence of FLAs from spring to summer months was seen in all genera, except for *Naegleria* in which the percentage of households positive was lower than it was in the spring and fall. The same authors concluded that there was no appreciable difference in detections across the years of the study (32). Generally, an increase in FLAs during the summer has been found in Oklahoma, Virginia and South Carolina waters (36, 37).

In this investigation, *Acanthamoeba* species were the most prevalent FLAs in the examined water samples. *Acanthamoeba* species were the most widely recognized opportunistic amphizoic protozoa in water (14, 24). These microorganisms have increasing therapeutic significance since some of them can deliver pathologies in people, for example, amebic encephalitis (37, 38), amebic keratitis and a sight threatening ulceration of the cornea (39, 40).

## Conclusion

Although the removal of FLAs through each of RSFS-DWTP and SSFS-DWTP was statistically significant (*P*=0.017), there was no significant difference (*P*=0.55) between the two different drinking water treatment plants for the removal of FLAs. The relatively high prevalence of *Acanthamoeba* spp. in the produced drinking water presented health hazards to consumers.
